# Transfer Learning for Improved Audio-Based Human Activity Recognition

**DOI:** 10.3390/bios8030060

**Published:** 2018-06-25

**Authors:** Stavros Ntalampiras, Ilyas Potamitis

**Affiliations:** 1 Music Informatics Laboratory, Department of Computer Science, Università degli Studi di Milano, via Comelico 39, 20135, Milan, Italy; 2 Technological Educational Institute of Crete, E. Daskalaki, Perivolia, 74100, Rethymno, Greece; potamitis@staff.teicrete.gr

**Keywords:** transfer learning, generalized audio recognition, multidomain features, hidden Markov model, echo state network

## Abstract

Human activities are accompanied by characteristic sound events, the processing of which might provide valuable information for automated human activity recognition. This paper presents a novel approach addressing the case where one or more human activities are associated with limited audio data, resulting in a potentially highly imbalanced dataset. Data augmentation is based on transfer learning; more specifically, the proposed method: (a) identifies the classes which are statistically close to the ones associated with limited data; (b) learns a multiple input, multiple output transformation; and (c) transforms the data of the closest classes so that it can be used for modeling the ones associated with limited data. Furthermore, the proposed framework includes a feature set extracted out of signal representations of diverse domains, i.e., temporal, spectral, and wavelet. Extensive experiments demonstrate the relevance of the proposed data augmentation approach under a variety of generative recognition schemes.

## 1. Introduction

Recognizing the activities performed by humans could provide invaluable information in the context of an automated machine. Human Activity Recognition (HAR) is particularly relevant under a series of application contexts, such as medical, military, and security. For example, having knowledge of the activities performed by a soldier in the combat zone may be extremely useful to optimize one’s strategy. In the same line of thought, monitoring the sequence of activities performed by patients suffering from hypertension could be beneficial towards providing effective treatment, medication, etc. Another important consideration is enhancing the quality of life for humans in terms of, e.g., safety, security, comfort, and home care.

Due to the recent explosion of sophisticated wearable devices able to capture a wide range of parameters [1,2,3,4], the majority of HAR literature is based on information coming from the integrated sensors, such as accelerometers, gyrometers, etc. Unlike that line of reasoning, the present work focuses on external microphone sensors and processes the acquired audio signals for extracting relevant characteristics and recognize the performed human activity. Audio-based situational awareness is already exploited in vocal assistants such as Google Home, Apple HomePod, and Amazon Echo. Even though currently such devices are focused on human speech, their functionality could be complimented towards recognizing human activities carried out in domestic environments.

At the same time, there is a substantial difference with respect to the recent Detection and Classification of Acoustic Scenes and Events challenges [5]; in those challenges, sounds represent soundscapes, which [6] are not necessarily indicative of human activities. Coming from the related literature [7,8], one of the most significant obstacles in fulfilling audio-based HAR (AHAR) is the fact that several classes are associated with a limited amount of data, thus researchers have to deal with highly imbalanced datasets. This work develops a methodology based on transfer learning [9], addressing exactly this problem.

AHAR literature is typically focused on the choice of a particular kind of activity $a_{i}$ belonging to an a priori given set of activities $A=\{a_{1},a_{2},\ldots ,a_{n}\}$, where *n* is the total number of activities. The final choice is based on the observation of characteristic features, or feature sequences, as the pattern recognition process tries to relate them to an activity $a_{i}\in A$. Moreover, it is implicitly assumed that the considered process is stationary over time, i.e., the distribution exhibited by each activity remains consistent. Finally, most existing works assume that A is bounded and includes the entire gamut of activities appearing during the operation of the system.

Stork et al. [8] used the well-known Mel-frequency cepstral coefficients (MFCC) to characterize each human activity, while the classifier was a random forest. They considered 22 human activities in a bathroom and kitchen context. An interesting paper focused on the detection of human activities in a biodiversity monitoring context is presented in [10]. The authors exploited various feature sets combined with an hidden Markov model classification scheme, while the dataset included sound events associated with four human activities. The work described by Galván-Tejada et al. [7] employed 16 statistical features of the MFCCs fed to a random forest classifier. The authors used a dataset of eight human activities performed in a domestic environment.

This work proposes an AHAR framework encompassing the following novel characteristics:

- the combinatorial usage of temporal, frequency, and wavelet acoustic features;
- the extensive exploration of the temporal evolution of these features by means of hidden Markov Models (HMMs) including both class-specific and universal ones; and
- a statistical transfer learning module specifically designed to address potential data imbalances.

Universal background modeling was explored, since it is an effective way to model imbalanced datasets [11]. This work exploits transfer learning for augmenting the data of the class(es) associated with limited data via transforming the feature spaces of closely (in the Kullback–Leibler sense) located classes. Transfer learning was selected as it is an effective tool in feature space transformation tasks involving audio signals [12,13,14], while learning the transformations among statistically-close feature spaces may be more effective than transforming ones with high Kullback–Leibler (KL) divergence. In this work, we are not interested in over- and/or under-sampling techniques for balancing the dataset as these might lead to model over-fitting [15,16]. As such, we wish to overcome the data scarcity problem by data augmentation.

Unlike the domain of face recognition and text analysis [17,18], this is the first time that transfer learning is explored in the AHAR research domain. Extensive experimentations, using the dataset and following the set-up of [7] demonstrate the efficacy of the proposed AHAR method. The considered human activities are the following: *brew coffee*, *cooking*, *use microwave oven*, *take a shower*, *dish washing*, *hand washing*, *teeth brushing*, and *no activity*.

This article has the following structure: Section 2 formalizes the problem. In sequence, Section 3 explains the proposed AHAR framework. After that, Section 4 provides an extensive description of the experimental protocol and the achieved results. Conclusively, Section 5 highlights the outcomes of this work.

## 2. Problem Formulation

In this paper, we suppose a single channel audio datastream, $y_{t}$, the duration of which is unknown. *y* may be emitted by various sources coming from the set $C=\{C_{1},\ldots ,C_{k}\}$, where *k* is the number of sound sources, each one associated with a specific human activity. It is further assumed that each source follows a consistent, yet unknown probability density function $P_{i}$ in stationary conditions, while at a specific time instance one sound source dominates (e.g., operating after a source separation framework [19]).

We assume that an initial training sequence $TS=y_{t},t\in \left[1,T_{0}\right]$ is available characterized by stationary conditions and containing supervised pairs $\left(y_{t}^{i},C_{i}\right)$, where $t\in [1,T_{0}]$ is the time instant and i∈[1,k]. The overall aim of a sound recognition system is to correctly recognize the incoming audiostream.

## 3. Transfer Learning-Based AHAR

This section described the proposed process towards data augmentation leading to audio-based HAR. In particular, the following subsections are dedicated to: (a) feature extraction; (b) probabilistic distance calculation; and (c) transfer learning based on Echo State Networks.

### 3.1. Acoustic Signal Parameterization

As our focus is not placed upon the feature extractions process, only a brief analysis of the employed feature sets is provided. Our intention is to acquire a multifaceted description of the audio signals, but more importantly to show that the applicability of the proposed transfer learning framework spans across all audio feature domains, i.e., *temporal*, *spectral*, and *wavelet*.

#### 3.1.1. Mel Frequency Cepstral Coefficients (MFCC)

This feature set has been employed in addressing a great variety of generalized sound recognition tasks [20]. Their basic purpose is to mimic the human auditory system to some extent. More specifically, during their computation, the nonlinearity of pitch perception as well as the nonlinear relationship between intensity and loudness are considered. In combination with their low computational cost, they have become the standard choice for many speech related tasks, such as language identification, emotion recognition, etc.

For their derivation, the signal is cut into frames of small duration based on the Hamming window technique. Afterwards, the short time Discrete Fourier Transform (DFT) is calculated for each frame using a predefined number of points. A triangular filter bank elaborates on the outcome of the DFT. Subsequently, the data are logarithmically spaced and the Discrete Cosine Transform is applied for exploiting its energy compaction properties as well as for feature decorrelation. Finally, we kept 13 feature coefficients along with the corresponding velocity. It should be mentioned that the first dimension of the MFCC vector, which comprises an excellent indicator of the energy of the signal, is considered as well. Hence, a feature vector of 26 dimensions is formed.

#### 3.1.2. MPEG-7 Audio Standard Low Level Descriptors (LLDs)

MPEG-7 audio protocol provides a set of standardized tools for automatic multimedia content description and offers a degree of “explanation” of the information meaning. It eases navigation of audio data by providing a general framework for efficient audio management. Furthermore, it includes a group of fundamental descriptors and description schemes for indexing and retrieval of audio data. Seventeen temporal and spectral descriptors which are useful for generalized sound recognition are utilized within the MPEG-7 audio standard. Several of them are quite simplistic (e.g., Audio Power) while others mainly target music processing (e.g., the ones that belong to the timbral group). The LLDs that may be proven effective as regards to the task of audio surveillance are:

- Audio Spectrum Centroid: The center of the log-frequency spectrum’s gravity is given by this descriptor. Omitting power coefficients bellow 62.5 Hz (which are represented by a single coefficient) makes able the avoidance of the effect of a non-zero DC component.
- Audio Spectrum Spread: The specific LLD is a measure of signal’s spectral shape and corresponds to the second central moment of the log-frequency spectrum. It is computed by taking the root mean square deviation of the spectrum from its centroid.
- Audio Spectrum Flatness: This descriptor is a measure of how flat a particular portion of the spectrum of the signal is and represents the deviation of the signal’s power spectrum from a flat shape. The power coefficients are taken from non-overlapping frames while the spectrum is typically divided into 1/4-octave resolution logarithmically spaced overlapping frequency bands. The ASF is derived as the ratio of the geometric mean and the arithmetic mean of the spectral power coefficients within a band.

#### 3.1.3. Perceptual Wavelet Packets (PWP)

Unlike the previous parameters, which come from either time or frequency domains, the specific feature set comes from the wavelet domain. The cornerstone of the particular transform is the fact that it is able to process non-stationary time-series at diverse frequency levels [21]. The work on generalized sound recognition presented by Ntalampiras et al. [10] motivated the integration of such features, as it demonstrated that acoustic parameters coming from diverse domains may be able to provide a complete picture of the structure of the audio signal resulting in improved performance.

The extraction of the PWP set is based on the Haar mother wavelet function. Initially, the spectrum of the audio signal is partitioned into critical bands similar to what done by the human auditory system [22,23]. Subsequently, a three-level wavelet packet transformation is applied onto each band. The extraction process ends by segmenting the coefficients, computing the area existing under the autocorrelation envelope and normalizing by the 50% of the size of each frame.

The PWP set is able to capture the variability exhibited by every wavelet coefficient within all critical frequency bands. As the audio signals associated with human activities demonstrate substantial differences in these bands, the usage of the PWP set could be advantageous. A MATLAB-based implementation of the specific set of acoustic features can be downloaded (for research intentions) [24]. The work reported by Ntalampiras et al. [25] offers a detailed description of the PWP set design and extraction process.

### 3.2. Identifying Statistically-Closely Located Classes

**Figure 1:**
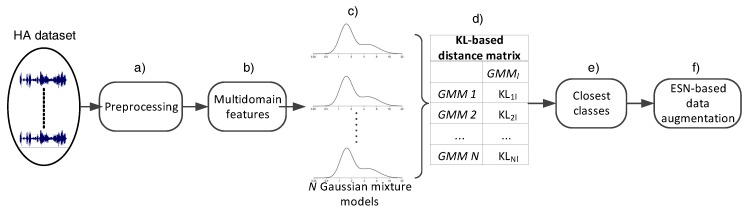
The logical flow of the proposed method. It includes: (**a**) signal preprocessing; (**b**) feature extraction; (**c**) GMM creation; (**d**) Kullback–Leibler divergence calculation as a distance metric; (**e**) identification of the closest models; and (**f**) data augmentation based on transfer learning.

As shown in Figure 1, a GMM, denoted as $G_{i},1<i<k$, is trained for estimating the distributions of each class $C_{i}$ using data in TS. Subsequently, the distances between the class(es) associated with limited data and the rest of of GMMs are computed. For the computation of each distance, a Monte Carlo approximation of the KL divergence is employed, the outcome of which is inversely analogous to the proximity among the involved distributions. For two distributions denoted as $p(F_{m}|\mu_{m},\sigma_{m},w_{m})$ and $p(F_{n}|\mu_{n},\sigma_{n},w_{n})$, the KL distance is defined as follows:

(1) $$KL\left(M||N\right)=\int p\left(F_{m}|\mu_{m},\sigma_{m},w_{m}\right)\log\frac{p(F_{n}|\mu_{n},\sigma_{n},w_{n})}{p(F_{m}|\mu_{m},\sigma_{m},w_{m})}dF_{n}$$

Due to the absence of a closed-form solution, the above formula is approximated by the empirical mean:

(2) $$KL\left(M||N\right)\approx \frac{1}{\omega }\sum_{i=1}^{n}\log\frac{p(F_{n}|\mu_{n},\sigma_{n},w_{n})}{p(F_{m}|\mu_{m},\sigma_{m},w_{m})}$$

This metric represents the distance quite accurately given that ω is sufficiently large. In our experiments, the number of Monte Carlo draws is ω=2000 [26]. In this work, KL divergence is used as a distance metric, thus symmetricity is required. However, in general the above defined quantity is not symmetric, i.e., the distance KL(M||N) may be different than KL(N||M). This burden was overcome by the usage of the following symmetrized form (also known as Jensen–Shannon divergence): Let KL(M||N) be denoted as $KL_{M}$ and KL(N||M) as $KL_{N}$. Then we get $KL\left(M||N\right)=KL\left(N||M\right)=KL_{M}+KL_{N}$. Finally, the closest model to the class associated with limited data is identified (denoted as $C_{d}$); the respective data are to be used for in the following stage.

### 3.3. ESN-Based Transfer Learning

Feature space transformation is essential for addressing the diversities existing in the feature distributions. We overcome the particular obstacle by learning an ESN-based transformation [27] which is suitable for capturing the potentially non-linear relationships associating different feature distributions. A multiple-input multiple-output (MIMO) transformation is learned using the training data of the class associated with limited data ($C_{l}$) and the one closest to it in the KL sense ($C_{d}$). ESN modelling, and in particular Reservoir Network (RN), was employed at this stage, as it is able to capture the non-linear relationships existing in the data. More precisely, RNs comprise a subcategory of ESNs which has shown excellent performance in many problems with diverse needs, e.g., saving energy in wireless communication [28], speech recognition [29], etc.

**Figure 2:**
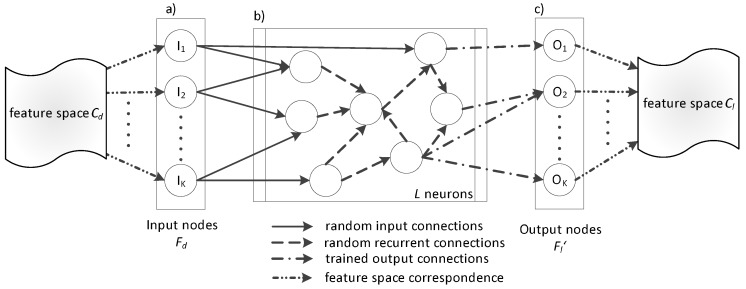
The Echo State Network used for feature space transformation (**a**) input layer, (**b**) reservoir layer, and (**c**) output layer) .

The typical topology of an RN is demonstrated in Figure 2. It is composed of neurons including non-linear activation functions with two possibilities: (a) connection with the input data (so-called input connections); and (b) connection to each other (so-called recurrent connections). Both are assigned randomly generated weights during the learning stage. It should be mentioned that these weights remain constant during the operation of the RN. Lastly, each output node holds a connection to a linear function.

The basic motivation behind reservoir computing lies behind the computational complexity of the back-propagation algorithm. During its application, the internal layers are not altered significantly, thus it is not included in RN learning. On the other hand, the output layer is associated with a linear problem of relatively low degree of perplexity. Nonetheless, the stability of the network is ensured by constraining the weights of the internal layers.

An RN includes parameters concerning the output weights, which are trained for reaching a given outcome, such as the features of a desired class achieve high values. Linear regression is employed to learn output weights, so-called read-outs in the literature. A detailed analysis of this process is provided at the works of Lukoševičius et al. [27] and Jaeger et al. [28].

In the following, we explain: (a) how the transfer learning RN (in the following denoted as tRN) learns the transformation from the feature space of class $C_{d}$ to that of class $C_{l}$; and (b) the exact way the transformation is employed.

#### 3.3.1. RN Learning

The tRN is used to learn the relationships existing in the features spaces of $C_{d}$ and $C_{l}$. We assume that an unknown system model is followed, which may be described as a transfer function $f_{RN}$.

$f_{RN}$ comprises an RN with *N* inputs and *N* outputs. Its parameters are the weights of the output connections and are trained to achieve a specific result, i.e., a $C_{l}$ feature vector. The output weights are learned by means of linear regression and are called read-outs since they “read” the reservoir state [27]. As a general formulation of the RNs, depicted in Figure 2, we assume that the network has *K* inputs, *L* neurons (usually called reservoir size), and *K* outputs, while the matrices $W_{in}\left(K\times L\right)$, $W_{res}\left(L\times L\right)$ and $W_{out}\left(L\times K\right)$ include the connection weights. The RN system equations are the following:

(3) $$\begin{array}{ccc} x(k) & = & f_{res}\left(W_{in}u\left(k-1\right)+W_{res}x\left(k-1\right)\right) \end{array}$$

(4) $$\begin{array}{ccc} y(k) & = & f_{out}\left(W_{out}\right)x\left(k\right), \end{array}$$

where u(k), x(k) and y(k) denote the values of the inputs, reservoir outputs and the read-out nodes at time *k*, respectively. $f_{res}$ and $f_{out}$ are the activation functions of the reservoir and the output nodes, respectively. In this work, we consider $f_{res}\left(x\right)=tanh\left(x\right)$ and $f_{out}\left(x\right)=x$.

Linear regression is used to determine the weights $W_{out}$,

(5) $$\begin{array}{ccc} W_{out} & = & arg\underset{W}{min}(\frac{1}{N_{tr}}{∥XW-D∥}^{2}+{\epsilon ∥W∥}^{2}) \end{array}$$

(6) $$\begin{array}{ccc} W_{out} & = & {\left(X^{T}X+\epsilon I\right)}^{-1}\left(X^{T}D\right), \end{array}$$

where XW and *D* are the computed vectors, *I* a unity matrix, $N_{tr}$ the number of the training samples while ϵ is a regularization term.

The recurrent weights are randomly generated by a zero-mean Gaussian distribution with variance *v*, which essentially controls the spectral radius SR of the reservoir. The largest absolute eigenvalue of $W_{res}$ is proportional to *v* and is particularly important for the dynamical behavior of the reservoir [27]. $W_{in}$ is randomly drawn from a uniform distribution [−InputScalingFactor, +InputScalingFactor], which emphasises/deemphasises the inputs in the activation of the reservoir neurons. It is interesting to note that the significance of the specific parameter is decreased as the reservoir size increases.

Here, $f_{RN}$ adopts the form explained in Equations (3) and (4) by substituting y(k) with $F_{l}$ and u(k) with $F_{d}$, where $F_{d}$ denotes a feature vector of class $C_{d}$ and $F_{l}$ a feature vector of class $C_{l}$.

#### 3.3.2. Application of $f_{RN}$

After learning $f_{RN}$, it may be thought as a MIMO model of the form:

(7) $$\left(\begin{array}{c} F_{l}^{1^{\prime }}\left(t\right)\\ F_{l}^{2^{\prime }}\left(t\right)\\ \vdots \\ F_{l}^{K^{\prime }}\left(t\right) \end{array}\right)=f_{RN}\left(\begin{array}{c} F_{d}^{1}\left(t\right)\\ F_{d}^{2}\left(t\right)\\ \vdots \\ F_{d}^{K}\left(t\right) \end{array}\right)$$

where the features $F_{d}^{1}\ldots ,F_{d}^{K}$ at time *t* are transformed using $f_{RN}$ to observations belonging to the $C_{l}$ class, i.e., features $F_{l}^{1^{\prime }}\ldots ,F_{l}^{K^{\prime }}$, where *K* denotes the dimensionality of the feature vector shared by both domains. It should be noted that *K* depends on the feature set, i.e., MFCCs, PWPs, and MPEG-7 LLDs.

### 3.4. Pattern Recognition of Human Activities

The last step concerning the categorization of novel sounds is recognizing the respective audio patterns. This work follows the path of the existing literature [30,31,32,33], including previous works of ours [34], thus we focus on modeling the temporal evolution of sound events, which may provide relevant discriminative information. To this end, we employ both class-specific and universal HMMs. In brief:

- *Class specific HMMs*: During this phase, we create one HMM to represent each sound class (using data associated with the specific class alone) and we follow the left–right topology, which is typically used by the community due to the nature of most sounds.
- *Universal HMM* [34,35]: During this phase, one HMM is created based on the entire training dataset while adapted versions of it are used to represent each sound class. In this case we use fully-connected (or ergodic) HMMs where every possible transition is permitted by the model which comprises a more appropriate choice given the variability of the entire dataset.

## 4. Experimental Set-Up and Analysis of the Results

This section includes: (a) a brief description of the dataset (a detailed analysis is available by Galván-Tejada et al. [7]); (b) the parameterization of the feature extraction, transfer learning, and classification modules; and (c) thorough comparative experimental results.

### 4.1. The Dataset

The corpus [36] employed in this work was taken from [7] and it was used in an identical way enabling a comparative study. It includes data associated with the following human activities: *brew coffee*, *cooking*, *use microwave oven*, *take a shower*, *dish washing*, *hand washing*, *teeth brushing*, and *no activity*. Aiming at a method with generic applicability, the recording devices have different specifications including mobile phones and operating systems. Overall, the dataset includes 1159 10-s audio clips, while each system variant was evaluated following 3-fold cross validation. The quantities of audio data per class of human activity are tabulated in Table 1.

It should be emphasized that we employed a real-world dataset recorded using various smart-phone devices, i.e., Lanix Ilium s600, LG G Pro Lite, iPhone 4, iPhone 3GS, and HTC One M7. These are equipped with heterogeneous system on chip technologies (Qualcomm Snapdragon 210 MSM8909, MediaTek MT6577, Apple A4 APL0398, Samsung S5PC100, and Qualcomm Snapdragon 600 APQ8064T) as well as operating systems (Android and iOS). With a focus on generalization, all sound events were captured in spatial environments with diverse characteristics in terms of reverberations, background noises, etc. including different home appliances. The interested reader is referred to [7] for more information on the database construction and recording protocol.

### 4.2. System Parameterization

#### 4.2.1. Feature Extraction

To extract the feature vector, each audio signal is framed into parts of 30 ms overlapping by 20 ms [37]. The FFT size is 512 and the hamming window type is used. Furthermore, we applied standard normalization methods (mean removal and variance scaling) onto $F_{v}$ as follows $F_{v}^{\prime }=\frac{F_{v}-\mu }{\sigma }$, where the statistical moments μ and σ are computed on the training set.

#### 4.2.2. ESN

The ESN [38] parameters were selected by means of exhaustive search based on the minimum reconstruction error criterion. The parameters were taken from the following sets: SR∈{0.8,0.9,0.95,0.99}, L∈{0, 500, 1000, 5000, 10,000}, and InputScalingFactor∈{0.1,0.5,0.7,0.95,0.99}.

#### 4.2.3. HMM

Torch implementation [39] of Gaussian Mixture Model (GMM) and HMM was used during the experimental phase. Left–right topology accompanied with GMMs of diagonal covariance matrices were employed. The thresholds with respect to *k*-means iterations, EM and Baum–Welch algorithms were set equal to 50, 25 and 0.001 between subsequent iterations.

Moreover the following sets of states were explored for the creation of the class-specific and universal HMMs, respectively:

- number of states: {3,4,5,6,7} and {5,6,7,8,9,10}, while
- number of Gaussian components: {2,4,8,16,32,64,128} and {64,128,256,512}

The model offering the maximum recognition accuracy is chosen. Finally, the KL divergence was computed on models composed of 16 modes. Early experimentations exploring with different number of Gaussians did not influence the overall performance significantly.

### 4.3. Experimental Results

The proposed TL data augmentation was applied as follows: the statistically-closest class was identified with respect to all the classes, and the ones associated with smaller quantities of data were augmented as described in Section 3.3. The final pairs were the following:

- *brew coffee–dish washing*,
- *cooking–use microwave oven*,
- *take a shower–teeth brushing*, and
- *no activity–hand washing*.

Towards a thorough assessment of the efficacy of the proposed TL-based data augmentation module, we experimented with four sound recognition systems: class specific HMM (cHMM), TL-based cHMM, universal HMM (uHMM), and TL-based uHMM. The respective confusion matrices are tabulated in Table 2. The achieved average recognition rates are 83.1%, 89.5%, 88.5%, and 94.6%, respectively. Average values over 50 iterations are reported. As we can observe, both classification approaches benefit from applying the TL-based data augmentation method. This carries significant meaning, since these perform classification from two different perspectives: the first one estimates the probability density function of each human activity independently from the rest, while the second one models the entire dataset holistically and, subsequently, tries to emphasize model components of each class. We can see that the recognition rates improve not only for classes associated with a limited amount of data but also for the rest since the refined models provide lower log-likelihoods when processing the previously misclassified sounds, thus resulting in correct classification. Conclusively, the best recognition rates are provided by the TL-enhanced universal HMM approach and the second one by the TL-enhanced class-specific HMMs. Both surpass the highest reported rate presented in the literature so far, i.e., 85.6% [7].

## 5. Conclusions

This paper presents an automatic framework for audio-based human activity recognition with enhanced recognition capabilities due a transfer learning data augmentation module. The efficacy of the proposed framework was revealed though a thorough experimental campaign. Its cornerstone is an ESN able to capture the non-linear relationships connecting the feature spaces of different classes of human activities. This way, the modeling accuracy of classes associated with limited amounts of data was improved, leading to boosted performance. We believe that such a technology can meaningfully enhance the performance of audio-based HAR systems leading to their full commercial exploitation. One straightforward application could be their incorporation in smart-home assistants, e.g., Google Home [40] , making them able to provide activity-aware suggestions and guidance.

Our future work includes the design and extensive evaluation of the data augmentation module to other classification tasks. In addition, we are interested in complimenting the present framework with a module providing resistance to evolving distributions. Clearly, $f_{RN}$ cannot be stationary over time as there is no one-on-one correspondence between data of two classes. We intent to develop a mechanism able to track such changes and update the data augmentation module.

## Figures and Tables

**Table 1:** The quantities of audio data per class of human activity.

Human Activity	10-Second Audio Clips
Brew coffee	245
Cooking	132
Use microwave oven	42
No activity	16
Taking a shower	428
Washing dishes	134
Washing hands	70
Brushing teeth	92

**Table 2:** The confusion matrix (in %) with respect to the class-specific and universal HMMs with and without the proposed TL data augmentation framework. The presentation format is the following: cHMM/cHMM-TL/uHMM/uHMM-TL. Average values over 50 iterations are shown. The achieved average recognition rates are 83.1%/89.5%/88.5%/94.6%. The highest rates are emboldened.

	Responded	*Brew Coffee*	*Cooking*	*Use Oven*	*Taking a Shower*	*Dish Washing*	*Hand Washing*	*Teeth Brushing*	*No Activity*
Presented									
*Brew coffee*		90.3/92/90.1/**95.7**	-/-/-/-	-/-/-/-	3.3/2.4/3/-	6.4/5.6/6.9/4.3	-/-/-/-	-/-/-/-	-/-/-/-
*Cooking*		-/-/-/-	88.5/93.3/91/**94.3**	11/6.7/8.1/5.7	-/-/-/-	-/-/-/-	-/-/-/-	-/-/-/-	0.5/0/0.9/-
*Use oven*		1.9/-/-/-	14.4/12.3/12.7/7.1	76.9/85.2/84.8/**92.9**	-/-/-/-	-/-/-/-	-/-/-/-	-/-/-/-	6.8/2.5/2.5/-
*Taking a shower*		-/-/-/-	-/-/-/-	-/-/-/-	91.7/93/92.2/**97.8**	-/-/-/-	-/-/-/-	5.9/4.9/5.7/2.2	2.4/2.1/2.1/-
*Dish washing*		12.4/12.1/12.1/9.6	-/-/-/-	-/-/-/-	3.7/3.6/3.6/-	83.9/84.3/84.3/**90.4**	-/-/-/-	-/-/-/-	-/-/-/-
*Hand washing*		-/-/-/-	-/-/-/-	-/-/-/-	3.6/-/-/-	-/-/-/-	78.9/92.5/92.5/**95.6**	-/-/-/-	17.5/7.5/7.5/4.4
*Teeth brushing*		-/-/-/-	-/-/-/-	-/-/-/-	14.9/10.8/11.4/4.8	-/-/-/-	-/-/-/-	80.6/87.9/87/**95.2**	4.5/1.3/1.6/-
*No activity*		6.7/-/-/-	-/-/-/-	-/-/-/-	-/-/-/-	-/-/-/-	19.3/12.6/13.7/4.9	-/-/-/-	74/87.4/86.3/**95.1**
